# Development of the treatment preference in myelodysplasia questionnaire for clinicians, carers, and patients

**DOI:** 10.1002/jha2.930

**Published:** 2024-05-28

**Authors:** Robert Morlock, Chun Fong, Francesco Castaldi, Taliesha Paine, Donna Collett, Anoop Enjeti

**Affiliations:** ^1^ Health Services Research YourCareChoice Ann Arbor Michigan USA; ^2^ Department of Clinical Haematology Austin Health Heidelberg Australia; ^3^ Specialty Care Otsuka Australia Pharmaceutical Pty Ltd Chatswood Australia; ^4^ Patient Insights Valeur Consulting Pty Ltd Sydney Australia; ^5^ Department of Haematology Calvary Mater Hospital Newcastle Australia

**Keywords:** hypomethylating agents, myelodysplastic syndromes, questionnaires, treatment preference

## Abstract

This study reports the development activities for the Treatment Preference Myelodysplasia Questionnaires (TPMQ) for clinicians (mTPMQ), carers (cTPMQ), and patients (pTPMQ). These tools are intended to evaluate treatment preferences for patients with myelodysplastic syndromes (MDS). This was a non‐interventional, cross‐sectional qualitative interview study consisting of interviews with clinicians, patients, and those caring for patients with MDS. All participants were located in Australia and data were collected from qualitative mixed‐method interviews composed of concept elicitation and cognitive debriefing related to initial drafts of the questionnaires. Fifteen individuals participated in interviews (five from each group). Based on the concept elicitation portion of interviews, concepts of importance were classified and reasons for treatment preference were documented. From cognitive debriefing, the questionnaires were generally deemed to be clear and easy to understand. Participant input from both concept elicitation and cognitive debriefing portions was used to revise initial drafts of the questionnaires. The mTPMQ, cTPMQ, and pTPMQ were developed with direct input from clinicians, patients, and caregivers to assess the key concepts of interest related to the preference for the treatment of MDS and are ready to be used and evaluated further in clinical trials.

## 1 INTRODUCTION

Myelodysplastic syndromes (MDS) are a heterogeneous group of myeloid neoplasms with bone marrow failure and inadequate hematopoiesis [1, 2]. MDS occurs preferentially in the elderly population with a median age at diagnosis of 70 years old and leads to an increased risk of developing acute myeloid leukemia (AML) [1]. Estimates of the incidence and prevalence of MDS vary; it has been suggested that between 60,000 and 500,000 people are living with MDS in the US [3, 4]. Globally, the incidence rate of MDS is 0.06–0.26 per 100,000 and incidence rates tend to increase with increasing age [5].

The approach to MDS treatment is typically stratified by factors identified by the International Prognostic Scoring System‐Revised and patient fitness, including age, performance status, and comorbidities [6]. Intermediate‐ to very‐high‐risk patients are recommended to receive induction chemotherapy with or without allogeneic stem cell transplant if they are fit enough to tolerate aggressive therapy, or hypomethylating agents (HMAs) if they are not [7]. In addition to these factors, patients and caregivers may have a preference for one treatment modality or another.

The injectable HMAs azacitidine and decitabine have been shown to be superior to best supportive care in improving cytopenias in intermediate to high‐risk patients [8, 9]. Both HMAs have multiple dosing options: azacitidine can be given as an injectable intravenously (IV) or subcutaneously (SC) over 7 days every 4 weeks and decitabine IV can be given in a 3‐day regimen every 6 weeks or a 5‐day regimen every 4 weeks [10, 11].

Injectable HMA regimens are commonly administered in a healthcare facility such as a hospital outpatient clinic, with patients traveling to the facility on consecutive days to receive treatment. However, the treatment regimen can be inconvenient; some dosing studies have noted that weekend administrations can be challenging for practitioners [12, 13]. Due to the inconvenience of the treatment regimen as well as clinical and demographic factors, it is hypothesized that some intermediate‐ to very‐high‐risk MDS patients who are HMA candidates may refuse therapy, discontinue therapy, or request temporary discontinuation of therapy [14, 15]. Patients report experiencing pain and discomfort after treatment with IV/SC HMAs and that the rigorous treatment regimen interferes with their social lives and daily activities [16]. Many patients have reported having to take time off of work and/or travel long distances to receive treatment [16], which can impose additional costs to patients and caregivers in the form of travel costs and lost wages. Zeidan et al. suggest that considering patients’ preferences regarding HMA treatment administration could help alleviate the burden that patients feel from undergoing the treatment regimen and encourage treatment adherence [17].

Preferences for different treatment options between patients, carers, and clinicians have not been evaluated extensively in MDS. An oral HMA has become available to treat MDS in some countries, including the United States, Canada, and Australia [18, 19]. The availability of oral alternatives may significantly impact patient, carer, and clinician choices. Given the different factors associated with potential MDS treatment options, it is important to understand the preferences of patients, carers, and clinicians regarding HMA treatments. Previously, studies have focused on the preferences of patients and caregivers, but not clinicians. Zeidan et al. developed a survey for patients or caregivers to assess preferences for the HMA therapy route [17], although the survey used in that study has not been validated empirically. Delmas et al. conducted telephone interviews with patients to understand their experiences and HMA therapy preferences [20] but did not develop a specific tool or preference set. Currently, there is no MDS‐specific tool exists that meets the patient‐reported outcome development recommendations of the U.S. Food and Drug Administration, the International Society for Quality of Life Research, or the International Society for Pharmacoeconomics and Outcomes Research.

## 2 OBJECTIVE

This study reports the development activities for the Treatment Preference Myelodysplasia Questionnaires (TPMQ) for clinicians (mTPMQ), carers (cTPMQ), and patients (pTPMQ). These tools are intended to evaluate the preference for injectable versus orally administered HMA treatment from each of these perspectives.

## 3 METHODS

### 3.1 Study type and patient population

This was a non‐interventional, cross‐sectional qualitative interview study consisting of interviews with clinicians treating patients with MDS, patients with MDS who were eligible for or were being/had been treated with azacitidine, and their carers. All participants were located in Australia. Data were collected from qualitative mixed‐method interviews composed of concept elicitation and cognitive debriefing.

Each site participated in a training session to discuss the study goal, inclusion and exclusion criteria, and recruitment procedures. Potential study participants were identified through office record review or during regularly scheduled office visits. Standardized central Human Research Ethics Committee (HREC)‐approved materials (such as the participant information sheet) were used by sites to screen potential participants and track enrollment into the study.

Sites were to identify, screen, and recruit patient, carer, and clinician participants. After the initial screening process, eligible participants (i.e., clinicians, carers, and patients) were sent a participant information sheet (PIS) and a participant informed consent form (PICF) for review. The PIS contained contact information for the study team and informed the participants that they could contact the team if they had any questions or wanted to request a hard copy of the informed consent form to be sent by standard mail. This study was approved by a human research ethics committee (HREC reference number 2021/ETH11640). An interview was scheduled once the participant and the authorized person obtaining the consent had signed the PICF. Prior to the interview, participants completed a background questionnaire (Supporting Information), to obtain their background demographic information.

Full details of inclusion and exclusion criteria can be found in the Supporting Information. To be included in the study, participants were required to be patients with MDS previously, currently, or soon to be treated with azacitidine, a carer, or a clinician treating patients who met the inclusion criteria. Participants were excluded from the study if they had a lower‐risk disease (IPSS of low‐ or intermediate‐ risk) or met any of the other exclusion criteria including cognitive or physical impairment, being part of a clinical trial with azacitidine, or being a relative of the research organization.

Interviews lasted approximately 60 min, took place by phone conference, and were moderated by experienced moderators/interviewers using a semi‐structured interview guide. All interviewers participated in training prior to carrying out any interviews. Training consisted of a review of the study's purpose and guidance on qualitative interview techniques. Interviews were carried out in two parts. During the first part of the interview (concept elicitation), participants were asked about MDS and treatment options to best understand the constructs of interest relative to their specific circumstances and preferences. The second part of the interview (cognitive debriefing) asked the participants to review a draft questionnaire to ensure the constructs of interest were captured and that the questions, instructions, and response options were well understood and appropriate to the respondents’ age, treatment experiences, preferences, and lifestyle. The cognitive debriefing interviews relied primarily on verbal probes about the interpretation of questions and recall strategies. Such probes were initially scripted and then expanded as needed by the interviewer. Interviews were audio recorded and transcribed verbatim, as described in the PIS and PICF, and de‐identified (e.g., names and locations were masked).

Based on the concept elicitation portion of interviews, concepts of importance were classified for clinicians, carers, and patients. Participant input from both concept elicitation and cognitive debriefing portions was used to revise initial drafts of the questionnaires to ensure the constructs of importance were captured and that the items, response options, and instructions were well understood. Modifications to the questionnaires based on participant feedback led to the final version of each questionnaire.

## 4 RESULTS

### 4.1 TPMQ for clinicians

Five clinician interviews were completed between June and August 2022. Of the 5 clinician participants, 60% (*n* = 3) were male and 40% (*n* = 2) were female. Sixty percent (*n* = 3) had practices in Newcastle, New South Wales (NSW), while 40% (*n* = 2) had practices in Heidelberg, Victoria (VIC). Sixty percent (*n* = 3) had practiced over 10 years, and 40% (*n* = 2) had practiced for less than 5 years. All had specialist qualifications and were licensed to practice as clinical hematologists in Australia. From the concept elicitation portion of the interviews, clinicians indicated several reasons for treatment preference, including efficacy, tolerance, manageability, patient preference, compliance, hesitancy to switch a patient's treatment, and the patient's response to treatment.

Cognitive debriefing resulted in revisions to the mTPMQ. Overall, clinicians indicated the directions, items, and response options were understandable and clear. The clinicians made a series of insightful comments that were helpful in terms of clarifying the questions and response options (e.g., add “(subcutaneous)” after “Azacitidine” and “(oral)” after “Decitabine/Cedazuridine”). Comments that significantly modified the questionnaire concerned splitting the last question from 1 to 2 questions, depending on which treatment the patient would be receiving. Initially, the clinicians answered one question on which factors influenced their decision regardless of the treatment selected. However, based on clinician comments, clinicians preferred more clarity and being directed to a question specifically related to the treatment choice selected. For example, if the clinician selected injectable, they would be directed to a question related to “Azacitidine (subcutaneous)” and if they selected the oral treatment they would be directed to a question for “Decitabine/Cedazuridine (oral).” Final concepts and response options for the mTPMQ are shown in Table 1.

**TABLE 1:** Concepts and response options from the three questionnaires.

	mTPMQ for clinicians	cTPMQ for carers	pTPMQ for patients
Treatment preference	Azacitidine (subcutaneous) Decitabine/ cedazuridine (oral) No preference	Injection Oral/tablets No preference	Injection Oral/tablets No preference
Strength of preference	Extremely strong Very strong Moderately strong Slightly strong Not at all strong	Very strong Fairly strong Not very strong	Very strong Fairly strong Not very strong
Reasons for stated preference	For either treatment: More efficacious Better tolerated Easier to manage Patient prefers Better patient compliance	For injections: More convenient and easier to manage Don't need to remind the patient to take his/her medication We benefit from more time in the clinic Easier to continue treatment Less emotionally distressing Prefer someone else gives the treatment Less costly For oral tablets: More convenient and easier to manage It means less time in the clinic Easier for the patient to take an oral tablet Less emotionally distressing Easier to continue treatment Less burdensome Easier to manage my schedule Less costly	For injections: More convenient and easier to manage I don't need to remember to take my medication I benefit from more time in the clinic Easier to continue treatment Less emotionally distressing Prefer someone else gives the treatment Less costly For oral tablets: More convenient and easier to manage It requires less time in the clinic Easier to take an oral tablet Less emotionally distressing Easier to continue treatment Less burdensome on my carer(s) Easier to manage my schedule Less painful and I don't have to have needles Less costly

Abbreviation: TPMQ, Treatment Preference Myelodysplasia Questionnaire (for clinicians [mTPMQ], carers [cTPMQ], and patients [pTPMQ]).

### 4.2 TPMQ for carers

A total of five carers were interviewed; 80% (*n* = 4) were female and 20% (*n* = 1) were male, with an average age of 77.2 years (minimum‐maximum: 71–81). Eighty percent (*n* = 4) were married to the patient. The 20% (*n* = 1) who were not married were widowed, and their relationship to the patient was as a lodger/friend. All carers were retired and lived in NSW. Sixty percent (*n* = 3) of the carers accompanied the patient to the clinic for injections. For the other 40% (*n* = 2), one patient was driven to the clinic by the Department of Veteran Affairs, and the other patient lived close to the hospital and drove themselves.

A number of factors influencing carers’ treatment preferences were identified during the concept elicitation portion of the interviews. These included convenience and manageability, the need to remind the patient to take the treatment, the time required to be in a clinic setting, the ease of patients taking and continuing the treatment, the amount of emotional distress, who is required to administer the treatment, the ease of managing their own schedule, and costs.

Cognitive debriefing revealed that, in general, carer participants thought the questionnaire captured the constructs of interest, the questions were clear, and the questionnaire was easy to answer. Modifications were made to the questionnaire as a direct result of carer participant comments (e.g., changed “We spend less time in the clinic” to “It means less time in the clinic for the patient”), study team discussions of the interviewer's comments and notes (e.g., added “It makes it easier for the patient to continue treatment”) and making items consistent between questionnaires. Concepts and response options of the final version are shown in Table 1.

### 4.3 TPMQ for patients

There were 5 patient participants; 80% (*n* = 4) were male and 20% (*n* = 1) were female, with an average age of 79.8 years (minimum–maximum: 74–85). Eighty percent (*n* = 4) were married, 20% (*n* = 1) were divorced; all were retired and lived in NSW.

During concept elicitation, patients reported many of the same factors influencing treatment preference that were reported by carers. Patients indicated that convenience and manageability of treatment, the time required to be in the clinic, the need to remember to take the treatment, the ease of taking and continuing treatment, the amount of emotional distress associated with a treatment, who administered treatment, the amount of pain associated with receiving treatment, the ease of managing their schedule, and costs were all factors. Additionally, patients noted that the burden of the treatment on their carers was also a factor.

From the cognitive debriefing interviews, patient‐participants generally thought the questionnaire captured concepts of interest and was easy to understand. Modifications were made to the questionnaire either as a direct result of patient participant comments (e.g., changed skip directions to “turn to page X, question X”), study team discussions of the interviewer's comments and notes, and making items consistent between questionnaires. Concepts and response options included in the final version are shown in Table 1.

## 5 DISCUSSION

Many studies have evaluated preferences for modes of medication administration. This is the first study to develop assessments for treatment preferences for HMA administration for clinicians, carers, and patients. Seeking input directly from those being treated, caring for those being treated, or overseeing treatment, respondents offered several factors that influenced the preference of treatment mode during the concept elicitation phase of questionnaire development. These concepts were included in the new assessments, increasing the likelihood of content validity of the tools. Cognitive debriefing indicated that respondents could respond to items and talk about all aspects assessed in the questionnaires in meaningful ways. Additionally, respondents were able to comprehend the instructions and response options on a categorical scale. Feedback from respondents who participated was valuable in creating the preference assessments for clinicians, carers, and patients.

The empirical validation of components of these tools will be carried out in a crossover trial where patients will experience both the injection and oral HMA treatments and then have the option to receive their treatment of choice (ClinicalTrials.gov NCT #05883956).

Existing MDS preference literature suggests that patients prefer an oral pill to IV/SC injections. The results of a patient/caregiver survey (*n* = 184, including 158 patients) revealed that most respondents (76.6%) were predicted to switch to an oral pill if it were available to them [17]. Telephone interviews with AML patients produced similar results: 71% preferred oral administration for HMAs [20]. There is limited data regarding physician preference.

Treatment failure among patients receiving HMA is common and can result in significant healthcare costs for those patients [3, 21]. Some studies suggest that low persistence with HMA therapy (which worsens over time) may be a factor in poor outcomes in real‐world treatment [22, 23]. A better understanding of the patient, carer, and clinician treatment preferences can help to understand why HMAs fail and the factors related to poor persistence. In addition to potentially improving treatment persistence, an oral HMA with similar efficacy and tolerability profiles to injectable HMAs could improve patients’ overall quality of life [20]. The development of clinician, caregiver, and patient versions of the TPMQ allows for more investigation into preferences regarding HMA administration and may further the understanding of the concordance or discordance of these preferences. These tools will also capture the strength of the preferences and the reasons behind them.

A strength of this study is the comprehensive development of all three versions using both concept elicitation and cognitive debriefing to ensure clarity and completeness. Additionally, this process was carried out in a representative patient population (elderly patient population with MDS disease and their carers). The inclusion of a diverse sample of clinicians is also a strength, given the paucity of existing data on clinician treatment preference.

A limitation of the questionnaires is that the tradeoff assessed is limited to oral versus subcutaneous administration. Although there is an IV‐administered form of azacitidine, in Australia, decitabine IV is not registered or available. The quantitative evidence supporting the validity of the mTPMQ, cTPMQ, and pTPMQ remains to be completed. Additionally, none of the participants had experience with both injectable and oral HMAs, and the study was conducted in a single country (Australia) and delivered in English with no language translation. Furthermore, the heterogeneity of the patient and carer ecosystems is extensive and this study was not able to take into account every possible scenario (e.g., younger patients, extended family caring for a patient, etc.). However, during the qualitative interviews, participants were given the opportunity to express preferences for themselves and others who may be in a similar situation, which informed the list of preferences in the assessments. Knowing that not all circumstances could be covered, the developers of the assessments added an open‐ended response to capture any preference not specifically identified in the questionnaire. During the review of the draft assessments, participants agreed that the open‐ended questions were important and provided the flexibility necessary for those in different circumstances than themselves.

The new patient and caregiver assessment, as designed, provides useful insights into patient and caregiver preferences when treatment decisions are considered. Ideally, the new assessments can be validated in a cross‐over trial where patients have experience with both injectable and oral HMA allowing the validity of the measures to be assessed by comparing preference to actual selection treatment.

## 6 CONCLUSIONS

The mTPMQ, cTPMQ, and pTPMQ were developed with direct input from clinicians, caregivers, and patients to assess the key concepts of interest related to the preference for the treatment of MDS to be used in further studies including clinical research to investigate patient, carer, and clinician preference for the treatment of MDS.

## AUTHOR CONTRIBUTIONS

Drs. Morlock, Castaldi, Paine, and Enjeti designed the study, developed the study protocol, and performed and reviewed analyses. Dr. Fong and Ms. Collett performed and reviewed analyses. All authors revised the manuscript, read it, and approved the final manuscript.

## CONFLICT OF INTEREST STATEMENT

Dr. Morlock reports personal fees from Otsuka during the conduct of the study and personal fees from Novartis, Heron Therapeutics, Replimune, Horizon Therapeutics, Syndax, Arthrosi, and Pfizer outside the submitted work.

Dr. Fong reports personal fees from Otsuka Australia Pharmaceutical during the conduct of the study, personal fees from AbbVie, Amgen, Beigene, Novartis, Pfizer, Servier, Bristol‐Myers Squibb, and grants and personal fees from Astellas and Jazz Pharmaceutical outside the submitted work.

Dr. Castaldi reports others from Otsuka Australia Pharmaceutical Pty Ltd during the conduct of the study and other fees from Otsuka Australia Pharmaceutical Pty Ltd outside the submitted work.

Dr. Paine is an employee of Otsuka Australia Pharmaceutical Pty Ltd.

Ms. Collett reports personal fees from Otsuka Australia Pharmaceutical Pty Ltd during the conduct of the study and personal fees from Otsuka Australia Pharmaceutical Pty Ltd outside the submitted work.

Dr. Enjeti reports others from Otsuka Australia during the conduct of the study, and personal fees from Otsuka Australia outside the submitted work.

## ETHICS STATEMENT

This study was reviewed and approved by a human research ethics committee (HREC reference number 2021/ETH11640) prior to commencement. All procedures involving human participants were conducted in accordance with the ethical standards outlined by the IRB, and informed consent was obtained from all participants prior to their involvement in the study.

## PATIENT CONSENT STATEMENT

Prior to participating in this study, all participants completed an informed consent process, wherein they were provided with detailed information about the study objectives, procedures, potential risks, and benefits. Participants were ensured confidentiality and their voluntary participation was explicitly acknowledged through the signing of informed consent forms.

## CLINICAL TRIAL REGISTRATION

The authors have confirmed clinical trial registration is not needed for this submission.

## Supporting information

Supporting Information

Supporting Information

Supporting Information

## Data Availability

All data presented in this manuscript are included within the text, figures, and supplementary materials. No additional datasets were utilized in the preparation of this study. Readers are encouraged to refer to the relevant sections of the manuscript for access to all supporting data.
