# Supplementary material for: Development of the treatment preference in myelodysplasia questionnaire for clinicians, carers, and patients
Source: EJHaem. 2024 May 28;5(3):535–40. doi: 10.1002/jha2.930 (PMC11182385; doi:10.1002/jha2.930)
Supplement: Supplementary file 1 — Supporting Information [file JHA2-5-535-s002.docx]

Participant Inclusion and Exclusion Criteria

### Inclusion Criteria

Participants must have satisfied all of the following criteria to be enrolled in the study:

1. Adult patients (≥ 18 years) with myelodysplastic syndrome meeting any one of the following criteria:
2. They are currently being treated with azacitidine or
3. They have recently been treated with azacitidine (no more than 3 months ago) or
4. They have been deemed eligible for treatment with azacitidine, but have not yet commenced treatment

OR

1. Primary carer of a patient meeting all of the inclusion criteria (i.e. a patient who meets criteria defined above in A).

OR

1. Clinician treating patients meeting all of the inclusion criteria (i.e. treats patients who meet criteria defined above in A).

### Exclusion Criteria

Participants who met any of the following criteria were excluded from participating in the study:

1. Patients meeting any of the following criteria are excluded:
2. They have lower risk disease (international prognostic scoring system [IPSS] low or intermediate-1 risk).^12^
3. Cognitive or physical impairment of a nature that makes it infeasible for them to effectively participate in a structured interview.
4. Deemed appropriate for stem cell transplantation and are being pre-treated or will be treated with azacitidine as a bridge to allogeneic transplantation
5. They were treated, are being treated, or will be treated with azacitdine as part of a clinical trial
6. They are a relative of an employee of the investigational clinic, sponsor, or research organization (e.g., Investigator, Coordinator, Technician, Interviewer).

OR

1. Carers meeting any of the following criteria are excluded:
2. They are a carer of a patient who meets any of the exclusion criteria listed above in A
3. They are a relative of an employee of the investigational clinic, sponsor or research organization (e.g., Investigator, Coordinator, Technician, Interviewer).

OR

1. Clinicians will be excluded from participating in the study if they are a relative of an employee of the sponsor or research organization (e.g., Investigator, Coordinator, Technician, Interviewer).
