# Supplementary material for: Development of the treatment preference in myelodysplasia questionnaire for clinicians, carers, and patients
Source: EJHaem. 2024 May 28;5(3):535–40. doi: 10.1002/jha2.930 (PMC11182385; doi:10.1002/jha2.930)
Supplement: Supplementary file 2 — Supporting Information [file JHA2-5-535-s003.docx]

Participant Background Questionnaires

**Clinician Background Questionnaire**

WE ARE CONDUCTING A RESEARCH STUDY IN WHICH YOU MAY BE ELIGIBLE TO PARTICIPATE. IF YOU ARE INTERESTED, THERE ARE A FEW CONFIDENTIAL QUESTIONS. YOU DO NOT HAVE TO ANSWER ANY QUESTIONS THAT YOU DO NOT WANT TO RESPOND TO, AND YOU MAY STOP COMPLETING THE QUESTIONNAIRE AT ANY TIME. ALL INFORMATION COLLECTED WILL BE KEPT CONFIDENTIAL. THIS SURVEY WILL BE DELETED AND NOT USED IF YOU DECLINE PARTICIPATION.

If you choose to participate in this study, this background questionnaire will be kept with other research records for this study. These records are accessible to our research staff and will not be shared with anyone else without your permission.

Are you willing to complete the background questionnaire?

O Yes

O No

[PROGRAMMER NOTE: if yes continue and if no end]

Please enter your Study ID: (sent by email or provided by the clinic)

QUESTIONNAIRE

1. What is your gender?

- Male
- Female
- Non-Binary

1. Where is your practice located?

City State

1. What is your advanced degree?

⭘ FRACP or equivalent

⭘ FRCPA or equivalent

⭘ Advanced trainee

1. How many years in practice? Be more specific:

⭘ < 5

⭘ 5-10

⭘ >10

1. About how many patients with Myelodysplastic Syndromes (MDS) treated with hypomethylating agents do you currently manage?
2. About how many patients with Myelodysplastic Syndromes (MDS) treated with hypomethylating agents do you see in a typical week?

**Carer Background Questionnaire**

WE ARE CONDUCTING A RESEARCH STUDY IN WHICH YOU MAY BE ELIGIBLE TO PARTICIPATE. IF YOU ARE INTERESTED, THERE ARE A FEW CONFIDENTIAL QUESTIONS. YOU DO NOT HAVE TO ANSWER ANY QUESTIONS THAT YOU DO NOT WANT TO RESPOND TO, AND YOU MAY STOP COMPLETING THE QUESTIONNAIRE AT ANY TIME. ALL INFORMATION COLLECTED WILL BE KEPT CONFIDENTIAL. THIS SURVEY WILL BE DELETED AND NOT USED IF YOU DECLINE PARTICIPATION.

If you choose to participate in this study, this background questionnaire will be kept with other research records for this study. These records are accessible to our research staff and will not be shared with anyone else without your permission.

Are you willing to complete the background questionnaire?

O Yes

O No

[PROGRAMMER NOTE: if yes continue and if no end]

Please enter your Study ID: (sent by email or provided by the clinic)

QUESTIONNAIRE

1. What is your gender?

- Male
- Female
- Non-Binary

1. What describes your relationship to the patient?

- Spouse
- Son or Daughter
- Other (please specify):

1. What is the year of your birth?
2. What is your marital status?

- Married / de facto
- Single (never married)
- Divorced
- Separated
- Widowed

1. Are you of Aboriginal or Torres Strait Islander origin?

- Yes
- No

1. Do you speak a language other than English at home?

- Yes
- No

If yes, please specify the language

1. What is your highest level of education?

- Postgraduate Degree
- Graduate Diploma or Graduate Certificate
- Bachelor’s degree
- Advanced Diploma or Diploma
- Certificate III/IV
- Year 12
- Year 10
- Certificate I/II
- Other (please specify)
- None of the above

1. Do any of the following apply to you? Select all that apply.

- Currently employed
- A student
- A Homemaker
- Retired
- Disabled, unable to work
- None of these

1. Employment Status: Are you currently…?

- Employed, working 35 hours per week or more
- Employed, working less than 35 hours per week
- Not employed, looking for work
- Not employed, not looking for work

1. How often do you take the patient to the clinic?

1. On average, when you take the patient to the clinic how much time does it take (e.g., ½ a day or 2 hours, etc.)?

1. When you take the patient to the clinic do you have any costs (for example, parking costs)? If so, please describe:

1. If you didn’t have to take the patient to the clinic, what do you think you would do instead (for example, go to work, work in the garden, etc.)?

**Patient Background Questionnaire**

WE ARE CONDUCTING A RESEARCH STUDY IN WHICH YOU MAY BE ELIGIBLE TO PARTICIPATE. IF YOU ARE INTERESTED, THERE ARE A FEW CONFIDENTIAL QUESTIONS. YOU DO NOT HAVE TO ANSWER ANY QUESTIONS THAT YOU DO NOT WANT TO RESPOND TO, AND YOU MAY STOP COMPLETING THE QUESTIONNAIRE AT ANY TIME. ALL INFORMATION COLLECTED WILL BE KEPT CONFIDENTIAL. THIS SURVEY WILL BE DELETED AND NOT USED IF YOU DECLINE PARTICIPATION.

If you choose to participate in this study, this background questionnaire will be kept with other research records for this study. These records are accessible to our research staff and will not be shared with anyone else without your permission.

Are you willing to complete the background questionnaire?

O Yes

O No

[PROGRAMMER NOTE: if yes continue and if no end]

Please enter your Study ID: (sent by email or provided by the clinic)

QUESTIONNAIRE

1. What is your gender?

- Male
- Female
- Non-Binary

1. What is the year of your birth? _
2. What is your marital status?

- Married / de facto
- Single (never married)
- Divorced
- Separated
- Widowed

1. Are you of Aboriginal or Torres Strait Islander origin?

- Yes
- No

1. Do you speak a language other than English at home?

- Yes
- No

If yes, please specify the language

1. What is your highest level of education?

- Postgraduate Degree
- Graduate Diploma or Graduate Certificate
- Bachelor’s degree
- Advanced Diploma or Diploma
- Certificate III/IV
- Year 12
- Year 10
- Certificate I/II
- Other (please specify)
- None of the above

1. Do any of the following apply to you? Select all that apply.

- Currently employed
- A student
- A Homemaker
- Retired
- Disabled, unable to work
- None of these

1. Employment Status: Are you currently…?

- Employed, working 35 hours per week or more
- Employed, working less than 35 hours per week
- Not employed, looking for work
- Not employed, not looking for work
