# Supplementary material for: Development of the treatment preference in myelodysplasia questionnaire for clinicians, carers, and patients
Source: EJHaem. 2024 May 28;5(3):535–40. doi: 10.1002/jha2.930 (PMC11182385; doi:10.1002/jha2.930)
Supplement: Supplementary file 3 — Supporting Information [file JHA2-5-535-s001.pdf]

Qualitative Interviews to Refine and Assess the Importance of Constructs, and Ensure the Readability of Patient, Carer, and Clinician Versions of the Treatment Preference in Myelodysplasia Questionnaire (TPMQ): pTPMQ, cTPMQ, and mTPMQ, Respectively.

Treatment Preference in Myelodysplasia Questionnaire: Patient

Instructions: Please answer the following questions given your experiences and preferences. There are no right or wrong answers.

1. Which treatment method did you prefer? *Select one response.*
   ○ Injection (turn to page 2, question 2)
   ○ Oral/tablets (turn to page 3, question 4)
   ○ No preference (**Stop. Please turn in your questionnaire**)

INJECTION QUESTIONS
(patient)

Instructions: Only answer the following questions if your treatment preference was for an **injection treatment.**

2. How strong is your preference for an **injection treatment?** *Select one response.*
   ○ Very strong
   ○ Fairly strong
   ○ Not very strong

3. Please rate the following reasons for your preference for an **injection treatment.** *Select one response per reason.*

	Very important	Fairly important	Not very important	Not at all important	Not applicable
More convenient and easier to manage	○	○	○	○	○
I don't need to remember to take my medication	○	○	○	○	○
I benefit from more time in the clinic	○	○	○	○	○
It feels less emotionally distressing	○	○	○	○	○
I prefer someone else to give the treatment	○	○	○	○	○
It makes it easier for me to continue treatment	○	○	○	○	○
There are less costs involved	○	○	○	○	○
Other (please specify): ________________________ ____ ____	○	○	○	○	○

Stop. Please turn in your questionnaire.

ORAL/TABLET QUESTIONS
(patient)

Instructions: Only answer the following questions if your treatment preference was for an **oral/tablet treatment.**

4. How strong is your preference for an **oral/tablet treatment?** *Select one response.*
   O Very strong
   O Fairly strong
   O Not very strong

5. Please rate the following reasons for your preference for an **oral/tablet treatment.** *Select one response per reason.*

	Very important	Fairly important	Not very important	Not at all important	Not applicable
More convenient and easier to manage	O	O	O	O	O
It requires less time in the clinic	O	O	O	O	O
It is easier to take an oral/tablet	O	O	O	O	O
It feels less emotionally distressing	O	O	O	O	O
It makes it easier for me to continue treatment	O	O	O	O	O
It is less burdensome on my carer(s)	O	O	O	O	O
It is easier to manage my schedule	O	O	O	O	O
It is less painful and I don't have to have needles	O	O	O	O	O
There are less costs involved	O	O	O	O	O
Other (please specify): ______________________	O	O	O	O	O

Stop. Please turn in your questionnaire.

Qualitative Interviews to Refine and Assess the Importance of Constructs, and Ensure the Readability of Patient, Carer, and Clinician Versions of the Treatment Preference in Myelodysplasia Questionnaire (TPMQ): pTPMQ, cTPMQ, and mTPMQ, Respectively.

Treatment Preference in Myelodysplasia Questionnaire: Carer

Instructions: Please answer the following questions given your experiences and preferences. There are no right or wrong answers.

1. Which treatment method did you prefer? *Select one response.*
   - O Injection (turn to page 2, question 2)
   - O Oral/tablets (turn to page 3, question4)
   - O No preference (**Stop. Please turn in your questionnaire**)

INJECTION PREFERENCE
(carer)

Instructions: Only answer the following questions if your treatment preference was for an **injection treatment.**

2. How strong is your preference for an **injection treatment?** *Select one response.*
   - O Very strong
   - O Fairly strong
   - O Not very strong

3. Please rate the following reasons for your preference for an **injection treatment.** *Select one response per reason.*

	Very important	Fairly important	Not very important	Not at all important	Not applicable
More convenient and easier to manage	O	O	O	O	O
I don't need to remind the patient to take his/her medication	O	O	O	O	O
We benefit from more time in the clinic	O	O	O	O	O
It makes it easier for the patient to continue treatment	O	O	O	O	O
It feels less emotionally distressing	O	O	O	O	O
I prefer someone else to give the treatment	O	O	O	O	O
There are less costs involved	O	O	O	O	O
Other (please specify): ______	O	O	O	O	O

Stop. Please turn in your questionnaire.

ORAL/TABLET QUESTIONS
(carer)

Instructions: Only answer the following questions if your treatment preference was for an **oral/tablet treatment.**

4. How strong is your preference for an **oral/tablet treatment?** *Select one response.*
   - O Very strong
   - O Fairly strong
   - O Not very strong

5. Please rate the following reasons for your preference for an **oral/tablet treatment.** *Select one response per reason.*

	Very important	Fairly important	Not very important	Not at all important	Not applicable
More convenient and easier to manage	O	O	O	O	O
It means less time in the clinic for the patient	O	O	O	O	O
It is easier for the patient to take an oral tablet	O	O	O	O	O
It feels less emotionally distressing	O	O	O	O	O
It makes it easier for the patient to continue treatment	O	O	O	O	O
It is less burdensome	O	O	O	O	O
It is easier to manage my schedule	O	O	O	O	O
There are less costs involved	O	O	O	O	O
Other (please specify): ________________________ ________________________ ________________________	O	O	O	O	O

Stop. Please turn in your questionnaire.

Treatment Preference Questionnaire: Medical (clinician)

Instructions: The following questions ask about your preference and choice of hypomethylating treatments decitabine/cedazuridine and azacitidine. The first 3 questions ask about your hypomethylating treatment preference, and the last 3 questions ask about the hypomethylating treatment that patient (ID NUMBER) will receive in the continuation phase of the trial.

1. All things considered which hypomethylating treatment do you prefer for this patient (ID NUMBER)? *Select one response.*
   ○ Azacitidine (*subcutaneous*)
   ○ Decitabine/cedazuridine (*oral*)
   ○ No preference

2. Which factors influenced your preference in Question 1? *Select all that apply.*
   ☐ I believe it is likely to be more efficacious for this patient
   ☐ I believe it is likely to be better tolerated by this patient
   ☐ I believe it is easier for me to manage this patient
   ☐ The patient prefers this treatment
   ☐ I believe it is better for this patient's compliance
   ☐ Other (please specify): ______________________________

3. How strong is your preference for the hypomethylating treatment you selected in Question 1? *Select one response.*
   ○ Extremely strong
   ○ Very strong
   ○ Moderately strong
   ○ Slightly strong
   ○ Not at all strong

4. Please indicate the actual treatment that patient (ID NUMBER) will be receiving in the continuation phase of the trial. *Select one response.*
   ○ Azacitidine (*subcutaneous*)…………….........*go to Q5*
   ○ Decitabine/cedazuridine (*oral*) ……………..*go to Q5*

   ○ No ongoing hypomethylating treatment …..*go to Q6*

5. Which factors influenced your decision? *Select all that apply.*
   ☐ I believe it is likely to be more efficacious for this patient
   ☐ I believe it is likely to be better tolerated by this patient
   ☐ The patient prefers this treatment
   ☐ I am reluctant to switch the patient's hypomethylating treatment again
   ☐ I believe it is better for this patient's compliance
   ☐ Other (please specify): ______________________________

   No further questions – questionnaire complete

6. Which factors influenced your decision? *Select all that apply.*
   ☐ The patient did not respond to hypomethylating treatment
   ☐ The patient did not tolerate hypomethylating treatment
   ☐ Other (please specify): ______________________________
